# Factors affecting willingness to comply with public health measures during the pandemic among sub-Sahara Africans

**DOI:** 10.4314/ahs.v21i4.17

**Published:** 2021-12

**Authors:** Obinna Nwaeze, Raymond Langsi, Uchechukwu L Osuagwu, Richard Oloruntoba, Godwin O Ovenseri-Ogbomo, Emmanuel K Abu, Timothy Chikasirimobi G, Deborah Donald Charwe, Bernadine Ekpenyong, Khathutshelo P Mashige, Piwuna Christopher Goson, Tanko Ishaya, Kingsley Agho

**Affiliations:** 1 County Durham and Darlington, National Health Service (NHS) Foundation, DL3 0PD, UK; o.nwaeze@nhs.net; 2 Health Division, University of Bamenda, Bambili, Cameroon; raylangsi@yahoo.com; 3 Translational Health Research Institute (THRI), School of Medicine, Western Sydney University, Campbelltown, NSW 2560, Australia; l.osuagwu@westernsydney.edu.au; 4 Curtin Business School, Curtin University, Bentley WA 6151, Australia; richard.oloruntoba@newcastle.edu.au; 5 Department of Optometry, University of Highlands and Islands, Center for Health Science, Old Perth Road, Inverness United Kingdom; godwin.ovenseri-ogbomo@uniben.edu; 6 Department of Optometry and Vision Science, School o fA llied Health Sciences, College of Health and Allied Sciences, University of Cape Coast, Ghana; eabu@ucc.edu.gh; 7 Department of Optometry and Vision Sciences, School o fp ublic health, Biomedical sciences and technology, Masinde Muliro University of Science and Technology, Kakamega Kenya; chikasirimobi@gmail.com; 8 Tanzania Food and Nutrition Center, P.O.Box 977 Dar es Salaam; mischarwe@yahoo.co.uk; 9 Department of Public Health, Faculty of Allied Medical Sciences, College of Medical Sciences, University of Calabar, Cross River State, Nigeria; bekpenyong@unical.edu.ng; 10 Westville Campus, African Vision Research Institute, Discipline of Optometry, University of KwaZu-lu-Natal, Durban 3629, South Africa; mashigek@ukzn.ac.za; 11 Department of Psychiatry, College of Health Sciences, University of Jos, Nigeria; piwunag@unijos.edu.ng; 12 Department of Computer Science, University of Jos, Nigeria; ishayat@unijos.edu.ng; 13 School of Health Sciences, Western Sydney University, Campbelltown, NSW 2560, Australia. K.Agho@westernsydney.edu.au

**Keywords:** Facemask, Handwashing, Self-isolation, Mitigation, Survey monkey, Pandemic, Lockdown, West Africa, Eastern Africa, South Africa, Nigeria

## Abstract

**Background:**

The unprecedented outbreak of coronavirus disease (COVID-19) drastically spread worldwide, resulting in extraordinary measures put in place in various countries including Sub Saharan Africa (SSA) countries.

**Objective:**

To assess the factors associated with compliance with the public health measures imposed by various SSA countries.

**Method:**

Cross sectional study using self-administered surveys distributed on social media platforms between April 18th and May 16th, 2020, corresponding with the mandatory lockdown period in most SSA countries. Multivariate analysis examined the associated factors.

**Results:**

The prevalence of hand hygiene, quarantine, self isolation practices, wearing of face mask and attending large gatherings during COVID-19 were 94%, 39%, 31%, 64% and 14%, respectively. In multivariate models, older age 49+ years: adjusted OR 2.13, 95%CI 1.22,3.71), females (OR 1.41,95%CI 1.03,1.93), Central African countries (OR 3.73,95%CI 2.02,6.87) were associated with wearing face mask. Living alone (aOR 1.52,95%CI 1.04,2.24) during the lockdown was associated with avoiding large gatherings including religious events. Female respondents (aOR 1.61, 95%CI 1.30, 2.00), married (aOR 1.71,95%CI 1.33,2.21) and unemployed (aOR 1.62,95%CI 1.25,2.09) SSAs were more likely to practice self-quarantine measures.

**Conclusion:**

The low prevalence of mitigation practices suggest the need for targeted education campaign programs to sensitise the population

## Introduction

Since December 2019, a newly identified coronavirus (Severe Acute Respiratory Syndrome Coronavirus 2, SARS-CoV-2) was found to cause an outbreak of coronavirus disease (COVID-19) in Wuhan, Hubei Province , China and spread to other countries by mid-January 2020 1. A combination of cases detected outside Wuhan, with the detection of infection in at least one household cluster and the documented infections in healthcare workers car risk of much wider spread of the disease 2. ing for patients with COVID-19, indicated human-to-human transmission of the virus and thus the

Globally, as of July 29, 2020, there have been 16,713,304 confirmed cases and in Africa, 789,226 cases have been confirmed 1. The first confirmed case in Africa was reported in Egypt on Feb 14, 2020, with this prompting African preparedness efforts^3^. Due to the lack of vaccines and effective pharmaceutical treatments at the time, nonpharmaceutical interventions (NPIs) were the most effective way to curb the spread of COVID-19^4^

The WHO interim guidance document of 7th March 2020 provided guidance to countries for responding to community transmission of COVID-19 through implementation of some public health measures appropriate to them^4^. The various public health measures adopted were at the national, community and individual levels. At the national level, governments introduced screening and quarantine of arrivals at airports and seaports as well as restriction of travels from countries highly affected by COVID-19 to finally suspending air travel into their countries 5, 6. At the community and individual levels, governments introduced several measures some of which were considered draconian. These measures included strict lockdown policy, staying at home to save life campaigns, travel and movement restrictions and prohibition of mass gatherings, regular hand washing under running water, frequent cleaning of surfaces with soap, or disinfectants 7, 8.

Combating misinformation and fake news about the pandemic formed part of the public health measures to minimize the spread of COVID -19 by educating the public on appropriate information for guidance on behaviors and practices^9^. In South Africa, a private firm Praekelt.org created a WhatsApp-based helpline that provided real-time date and automated responses in numerous languages to educate and sensitize people^10^.

In spite of the good intentions of the governments, there were factors militating against citizens' compliance to these measures. Central to this was economic hardships resulting from these enforced restrictions. This appeared most impractical in densely populated informal settlements and in economies largely dependent on informal trading. Religious practices, misinformation, and incidents of unrest were other factors implicated in affecting compliances^11^. In another research article, knowledge was quoted to be a prerequisite for establishing prevention beliefs, forming positive attitudes, and promoting positive behaviours, and that individuals' cognition and attitudes towards disease affect the effectiveness of their coping strategies and behaviours to a certain extent 12. This research was designed to assess the adherence to public health measures adopted in selected African countries on COVID-19 as well as to broadly evaluate the factors that influenced compliance to these measures in order to plan appropriately for the future. Findings from this study will enable public health researchers and policy makers to target sub-population not willing to comply with public health measures put in place by the respective SSA governments to contain and minimise the spread of COVID-19 infection in this region.

## Methodology

### Study population and design

This cross-sectional survey was conducted between the months of April and May 2020. During this time, most African countries were under the mandatory lockdown as implemented by their various governments. As it was not feasible to perform nationwide community-based sample survey during this period, the data were obtained electronically via survey monkey. A structured validated and pretested questionnaire was posted on social media platforms - Facebook and WhatsApp – to facilitate a better response. These platforms were chosen because they reflect the most commonly used social media platforms the locals in the participating countries engage with. Emails were also used. The questionnaire included a brief overview of the context, purpose, procedures, nature of participation, privacy and conidentiality statements and notes to be filled out.

The respondents were African nationals from different Sub-Sahara African countries living in their countries of origin or overseas including Ghana, Cameroon (only distributed to the English-speaking regions), Nigeria, South Africa, Tanzania, Kenya, Uganda and other countries which are shown in the Supplementary Figure. To be eligible for participation, respondents had to be aged 18 years or older and be able to provide online consent.

### Sample size determination

The study assumed a proportion of 50% of the population since similar study had not been carried out in Africa and with a desired precision of 2.5% and 5% significance level for a two-sided test. Assuming a non-response rate of 20%, the final sample size was calculated to be 1921 respondents, which was adequate to detect statistical differences in the analysis of this online cross-sectional study on COVID -19 in Africa. However 1801 respondents participated by completely answering questions on compliance to with public health measures during the COVID-19 pandemic.

### Survey Questionnaire and study factors

The questionnaire used in this study is presented as Supplemenary table 1. The survey tool for the COVID-19 was developed based on the guidelines from the WHO for clinical and community management of COVID-19^1^,^5^. The questionnaire was adapted with minor modifications to suit this study's objective. A pilot study was conducted to ensure clarity and understanding as well as to determine the duration for completing the questionnaire prior to dissemination. The questionnaire consisted of 53 items divided into four sections (demographic characteristics, knowledge, perception and practice). The demographic variables included questions on age, gender, marital status, education, employment and religion.

**Table 1:** Socio-demographics, knowledge and compliance of study sample (n=1801, except where indicated)

*Demographic Variables*	Frequency(n)	Percentage (%)
**Region of residence, n=1777**		
Africa	1,644	92.52
Diaspora	133	7.48
**Sub-region, n=1747**		
West Africa	982	56.21
East Africa	181	10.36
Central Africa	221	12.65
Southern Africa	363	20.78
**Age category (years), n=1773**		
18–28	676	38.13
29–38	482	27.19
39–48	390	22
≥49	225	12.69
**Sex, n=1774**		
Males	993	55.98
Females	781	44.02
**Employment status, n=1782**		
Employed	1,189	66.72
Unemployed	593	33.28
**Marital Status, n=1778**		
Married	790	44.43
Not married	988	55.57
**Religion, n=1779**		
Christianity	1,571	88.31
Others	208	11.69
**Educational status, n=1782**		
Postgraduate	592	33.22
Bachelor	971	54.49
Primary/Secondary	219	12.29
**Occupation, n=1688**		
Non-health care sector	1,300	77.01
Health care sector	388	22.99
**Household factor**		
**Do you live alone during COVID-19,** **n=1781**		
No	1,454	81.64
Yes	327	18.36
**Number living together, n=1557**		
< 3 people	452	29.03
4–6 people	802	51.51
6+ people	303	19.46
***COVID-19 -related knowledge***		
**Knowledge of origin/transmission**		
Inadequate	1,318	73.18
Adequate	483	26.82
**Knowledge of Symptoms**		
Inadequate	1,154	64.08
Adequate	647	35.92
**Perception of risk**		
Low	940	52.19
High	861	47.81

### Outcome variables

Six questions addressed willingness to comply with COVID-19 public health measures. These questions are the subject of this paper, and their wordings were as follows: “During COVID-19, government authorities might request co-operation from the public in a number of ways. Please indicate ...”

1 Are you currently or have you been in (domestic/home) quarantine because of COVID-19?

2 Are you currently or have you been in self-isolation because of COVID-19?

3 In recent days, have you worn a mask when leaving home?

4 In recent days, have you been washing your hands with soap and running water for at least 20 seconds each time?

5 In recent days, have you gone to any crowded place including religious events?

All responses except willingness to quarantine because of COVID-19 were coded on a five-point Likert-scale. Response options for all questions were ‘Always’, ‘Not Sure’, ‘Not at all’, ‘Rarely’ and ‘Sometimes’. In addition, willingness to quarantine because of COVID-19 responses were coded as ‘yes’ and ‘no’.

### Ethics

The study adhered to the principles of the 1967 Helsinki declaration (WMA, 2013) and the protocol was approved by the Human Research Ethics Committee of the Cross River State Ministry of Health, Nigeria (number: CRSMOH/RP/REC/2020/116). Participation was anonymous and voluntary. Informed consent was obtained from all participants prior to commencement of the study and after the study protocol has been explained. Participants consented to voluntarily participate in this study by answering either a ‘yes’ or ‘no’ to the question inquiring whether they voluntarily agree to participate in the survey. A ‘no’ response meant that the participants could not progress to answering the survey questions and were excluded from the study.

### Statistical analysis

Data analysis was performed using Stata version 14.1 (Stata Corp. College Station United States of America). Categorical variables were presented as frequencies and percentages. This was followed by estimation of the prevalence and 95% confidence intervals (CI) of each willingness to comply with COVID-19 public health measures.

The five-point Likert-scale response used in the question module were dichotomized, such that responses of ‘Always’ were coded as ‘1’ and all other responses as 0. This was done to aid epidemiological interpretations and to describe the type of outcome under study (prevalence study and odds ratios). Additionally, it is very difficult to determine normality from a Likert-scale In addition, quarantine because of COVID-19 responses were coded as 1 for ‘yes’ and 0 for ‘no’.

Univariable and Multiple logistic regression using a stepwise backwards model was used in order to identify the factors significantly associated with willingness to comply with health measures during COVID-19. All variables with statistical significance of p<0.05 were retained in the final model.

## Results

Of the 1801 respondents (males, n = 993, 56%) that completed the online questionnaire, about half (52.2%) were from West Africa and over 65% were aged below 39 years. Table 1 shows the demographic characteristics of the respondents as well as their knowledge of the origin/transmission of the disease, its symptoms and compliance with government regulations to prevent the spread of the infection. Respondents were mostly from Nigeria, Ghana, South Africa, Kenya, Cameroon and Tanzania (Supplemnetary figure). Knowledge of COVID-19 origin/mode of transmission and its symptoms were inadequate in more than two-thirds (73.2%) and 64.5% of the respondents, respectively. A significant proportion (52.0%) had a low risk perception of contracting COVID-19. With regards to compliance, majority (73.5%) reported adherence to each of the government prescribed measures to control the spread of infection with respect to avoiding crowded places (86%) during the lockdown and practice of hand hygiene (94.0%).

### Factors associated with the attendance to of large gatherings and use of facemasks in sub-Saharan Africa

Table 2 presents the unadjusted (OR) and adjusted odd ratios (aOR) for the factors associated with attending large gathering and use of face masks. Respondents living in the Central African region (OR 3.33, 95% CI 2.34, 4.75), those married (OR 1.39, 95% CI 1.06–1.84), those with university education (OR 1.42, 95% CI 1.04, 1.94) and those who live alone (OR 1.62, 95% CI 1.17, 2.23) were more likely to comply with the regulation on avoiding large crowds (including religious gatherings) while those aged 39 – 48years (OR 0.52, 95% CI 0.34, 0.76) and non-Christians (OR 0.46, 95% CI = 0.27, 0.79) were less likely to comply with this directive. The respondents who had a perceived high-risk of contracting COVID-19 (OR 1.45, 95% Cl 1.11, 1.190) were more likely to avoid large gatherings compared to those with low perception of risk. After adjusting for the potential confounders, respondents that lived alone during the pandemic (aOR 1.52, 95%CI 1.04, 2.24), and those that reported high perception of risk of contracting the infection (aOR 1.27, 95%CI 1.05, 1.55) were more likely to avoid large gatherings during the lockdown.

**Table 2:** Unadjusted (OR) and adjusted odd ratios (aOR) for factors associated with attending large gatherings, regular handwashing, self-quarantine and isolation during the lockdown. The 95% confidence intervals (CI) of the odd ratios are also shown

	Attended large gatherings	Worn mask in recent days					
Sociodemographic	OR	95%CI	aOR	95%CI	OR	95%CI	aOR	95%CI
**Region of** **residence**								
Local	1.00	-- --	--	--	1.00	-- --	--	--
Diaspora	1.51	0.96, 2.39		--	1.54	**1.04,** **2.29** --	--	
**Age category** **(years)**								
18–28	1.00	----	1.00	--	1.00	--	1.00	--
29–38	0.994	0.72, 1.36	0.94	0.65, 1.36	0.95	0.74, 1.20	0.69	0.48, 1.00
39–48	**0.52**	**0.34, 0.76**	**0.52**	**0.33, 0.82**	1.29	0.99, 1.68	1.34	0.88, 2.04
49+	**0.34**	**0.19, 0.60**	**0.27**	**0.14, 0.51**	**1.64**	**1.17,** **2.28**	**2.13**	**1.22,** **3.71**
**Sex**								
Males	--	--	--	--	1.00		1.00	--
Females	--	--	--	--	**1.60**	**1.31,** **1.95**	**1.41**	**1.03,** **1.93**
**Employment** **status**								
Employed	--	--	--		1.00	--	--	--
Unemployed	--	--	--		**0.76**	**0.62,** **0.93** --	--	
**Marital status**								
not married	1.00	--	--		1.00	--	--	--
Married	1.39	**1.06, 1.84**	--	--	**0.79**	**0.65,** **0.96** --	--	
**Sub-region**								
West Africa	1.00				1.00		1.00	
East Africa	1.53	0.97, 2.39	--	--	**2.69**	**1.86,** **3.89**	**2.37**	**1.37,** **4.10**
Central Africa	**3.33**	**2.34, 4.75**	--	--	**6.15**	**4.00,** **9.43**	**3.73**	**2.02,** **6.87**
Southern Africa	1.07	0.73, 1.57	--	--	**1.77**	**1.38,** **2.29**	**1.93**	**1.29,** **2.89**
**Religion**								
Christianity	1.00		1.00		1.00			
Others	0.46	0.27, 0.79	0.50	0.27, 0.93	1.11	0.82, 1.50		
**Educational status**								
Postgraduate	1.00				1.00			
Bachelor	**1.42**	**1.04, 1.94**	--	--	0.95	0.76, 1.17		
Primary/Secondary	1.21	0.76, 1.94	--	--	1.16	0.84, 1.62		
**Occupational status**								
Non-health care sector					1.00			
Health care sector	--			0.85	0.68, 1.08	--		
**Household factor**								
**Do you live alone** **during COVID-19**								
No	1.00			1.00			1.00	
Yes	**1.62**		**1.17, 2.23**		**1.52**	**1.04, 2.24**	0.87	0.68, 1.11
Number living together								
< 3 people					1.00			
4–6 people	-		--		0.99	0.77, 1.24 --		--
6+ people	--		--		1.04	0.77, 1.42 --		--
**COVID-19 -related** **knowledge**								
**Knowledge of** **origin/transmission**								
Inadequate					1.00			
Adequate	--			1.25	1.00, 1.56 --		--	
**Knowledge of** **Symptoms**								
Inadequate	--				1.00			
Adequate	--			1.04	0.85, 1.28 --	--		
**Perception of risk**								
Low	1.00				1.00			
High	**1.45**		**1.11, 1.90**		**1.27**	**1.05,** **1.55** --		--

Bolded are significant differences with 95% confidence intervals of odd ratios that does not include 1.00. Values are derived from stepwise regression model with empty cells representing variables not included in the final model.

In the unadjusted analysis, compliance with the recommendation to wear facemask when going out was associated with older age (49 years and over), living in diaspora, female sex, and respondents from the Eastern, Central and Southern African region. (Table 2). After adjusting for the potential cofounding variables, all the aforementioned factors except for place of residence (diaspora) remained significantly associated with the use of facemask in this cohort.

### Factors associated with practicing regular handwashing, self-quarantine and self isolation on recommendation among sub-Saharan Africans

Table 3 presents the unadjusted and adjusted odd ratio for factors associated with practicing regular handwashing, self-quarantine and self-isolation during the lockdown. The table shows that compliance with the practice of hand washing was significantly associated with increasing age with 14.2 folds (95% CI of OR 3.42,57.57) increase in the odds of hand washing among older respondents (49 years and above) compared to younger ones (18–28years). This association was lost after adjusting for potential cofounders. Central Africans (aOR 0.30, 95%CI 0.15, 0.57), those who were unemployed (aOR 0.30 95%CI 0.21,0.51) and respondents that had adequate knowledge of COVID-19 origin/transmission (aOR 0.48, 95%CI 0.31, 0.76) were less likely to practice hand hygiene compared to West Africans, the employed and those that demonstrated inadequate knowledge. Being female (aOR 1.61, 95% CI 1.30, 2.00), being married (aOR 1.71, 95% CI 1.33,2.21) and being unemployed (aOR 1.62, 95% CI 1.25,2.09) were associated with more likelihood of practising quarantine measures.

**Table 3:** Unadjusted (OR) and adjusted odd ratio (aOR) for factors associated with practicing regular hand washing, self-quarantine and isolation during the lockdown. The 95% confidence intervals (CI) of the odd ratios are also shown

Socio-demographic	Practiced Hand washing				Practiced quarantine				Self isolation			
	OR	95%CI	aOR	95%CI	OR	95%CI	aOR	95%CI	Odds ratio	95%CI	aOR	95%CI
**Region of residence**												
Local	1.00				1.00				1.00			
Diaspora	0.61	[0.33, 1.14]			0.70	[0.48, 1.02]			**1.37**	**[1.95, 1.97]**	--	--
**Age category (years)**												
18–28	1.00				1.00				1.00		1.00	
29–38	**3.09**	**[1.84, 5.19]**			**0.51**	**[0.40, 0.64]**			**0.48**	**[0.37, 0.61]**	**0.67**	**[0.50, 0.88]**
39–48	**6.03**	**[2.88, 12.63]**			**0.39**	**[0.30, 0.51]**			**0.31**	**[0.23, 0.41]**	**0.51**	**[0.36, 0.72]**
49+	**14.02**	**[3.41, 57.57]**			**0.36**	**[0.26, 0.50]**			**0.26**	**[0.18, 0.39]**	**0.42**	**[0.28, 0.64]**
**Sex**												
Males	1.00				1.00		1.00		1.00			
Females	1.12	[0.75, 1.68]			**1.71**	**[1.41, 2.07]**	**1.61**	**[1.30, 2.00]**	**1.29**	**[1.06, 1.58]**	--	--
**Employment status**												
Employed	1.00		1.00		1.00		1.00		1.00			
Unemployed	**0.25**	**[0.16, 0.38]**	**0.3**	**[0.21,0.51]**	**2.36**	**[1.93,2.89]**	**1.62**	**[1.25,2.09]**	**2.33**	**[1.90, 2.87]**	-	
**Marital status**												
Not married	1.00				1.00		1.00		1.00		1.00	
Married	**0.31**	**[0.19, 0.51]**	-	-	**2.29**	**[1.88, 2.80]**	**1.71**	**[1.33, 2.21]**	**3.03**	**[2.43, 3.77]**	**2.00**	**[1.51, 2.66]**
**Sub-region**												
West Africa	1.00		1.00		1.00		1.00		1.00			
East Africa	1.04	[0.51, 1.94]	0.62	[0.30, 1.29]	1.02	[0.72, 1.39]	0.96	[0.68, 1.37]	0.96	[0.68, 1.35]		
Central Africa	0.69	[0.40, 1.18]	**0.30**	**[0.15, 0.57]**	0.86	[0.63, 1.17]	**0.61**	**[0.44, 0.85]**	0.98	[0.71, 1.35]		
Southern Africa	1.52	[0.85, 2.71]	1.36	[0.73, 2.56]	1.12	[0.88, 1.43]	0.97	[0.74, 1.26]	1.18	[0.92, 1.53]		
**Religion**												
Christianity	1.00				1.00				1.00			
Others	0.61	[0.36, 1.04]	-	-	1.06	[0.79, 1.42]	-	-	1.31	[0.97, 1.77]		
**Educational status**												
Postgraduate	1.00				1.00				1.00			
Bachelor	**0.39**	**[0.22, 0.68]**	-	-	**1.48**	**[1.19, 1.84]**	-	-	**1.66**	**[1.31, 2.10]**		
Primary/Secondary	**0.23**	**[0.12, 0.44]**	-	-	**3.27**	**[2.38, 4.52]**	-	-	**3.27**	**[2.35, 4.53]**		
**Occupational status**												
Non health care	1.00				1.00				1.00			
Health care	0.94	[0.58, 1.51]	-	-	1.04	[0.83, 1.31]	-	-	1.02	[0.80, 1.30]		
**Household factors**												
**Do you live alone during** **COVID-19**												
No	1.00				1.00				1.00			
Yes	0.85	[0.52, 1.38]	-	-	1.00	[0.79, 1.28]	-	-	**1.63**	**[1.28, 2.09]**		
Number living together												
< 3 people	1.00				1.00		1.00		1.00			
4–6 people	1.20	[0.76, 1.90] -	-	-	1.03	[0.81, 1.31]	1.06	[0.82, 1.36]	1.00	[0.78, 1.29]		
6+	1.76	[0.91, 3.40]	-	-	1.32	[0.98, 1.77]	**1.40**	**[1.03, 1.91]**	1.33	[0.98, 1.82]		
**COVID-19 -related knowledge**												
**Knowledge of origin/transmission**												
Inadequate		1.00		1.00		1.00				1.00		
Adequate	**0.53**	**[0.35, 0.79]**	**0.48**	**[0.31, 0.76]**	**1.28**	**[1.04, 1.58]**	-	-	**1.44**	**[1.16, 1.80]**		
**Knowledge of Symptoms**												
Inadequate	1.00				1.00				1.00			
Adequate	1.03	[0.69, 1.55]	-	-	0.98	[0.80, 1.19]		-	**1.23**	**[1.00, 1.51]**		
**Perception of risk**												
Low	1.00				1.00				1.00		1.00	
High	1.26	[0.85, 1.86]	- -		1.05	[0.87, 1.26]	- -	-	**1.25**	**[1.02, 1.53]**	**1.26**	**[1.03, 1.56]**

Bolded are significant differences with 95% confidence intervals of odd ratios that does not include 1.00. Values are derived from stepwise regression model with empty cells representing variables not included in the final model.

Although education level was associated with observing quarantine and isolation measures, in the unadjusted analysis, the adjusted odds ratio did not show any significance.

## Discussion

This paper evaluated the public health measures at the individual and community levels enforced by African governments and considered the factors associated with compliance with these measures. The measures identified in this paper included personal measures like hand hygiene/hand washing, the use of face masks, physical and social distancing such as avoiding large crowds/mass gathering, isolation and quarantine. Respondents in SSA demonstrated a high level of compliance with avoiding crowded places, wearing of face masks and regular hand washing but varied between countries. Fewer respondents complied with the recommendation to self-isolate during the pandemic but knowledge of COVID-19 origin/mode of transmission and symptoms of the disease were inadequate. The factors associated with compliance with mitigation practices were age, marital status (being married), sex (female), central African residency as well as having adequate COVID-19 related knowledge and perceived high risk of contracting the infection.

The differences in compliance rate among SSA countries may suggest a direct link between the varying degrees of strictness of lockdown measures, sensitisation of the citizens and education, especially to the vulnerable groups. For example, there was a widespread reference to varying degrees of lockdown across SSA countries, such as ‘total lockdown’ and ‘partial lockdown,’ or ‘tight lockdown’ and ‘loose lockdown.’ Similar control measures were also important in successfully controlling SARS-CoV in 2003 and was substantially aided by important differences in the transmission dynamics of SARS-CoV compared with SARS-CoV-2^13^. As there is currently no effective pharmacological interventions or vaccines available to treat or prevent COVID-19, nonpharmacological public health measures such as isolation, social distancing, and quarantine remain the only effective ways to respond to the outbreak^14^. To discuss measures of controlling spread it is worth noting an established mode of transmission for COVID-19, particularly human-to-human transmission. This kind of transmission has been recognised with the major mode of respiratory tract transmission via droplets and indirectly from fomites and to a lesser extent via aerosols^15^.

The respondents in this study who were unemployed were more likely to self-isolate and quarantine but less likely to practice handwashing relative to those who were employed. A major component of the government efforts towards containing the spread is self-quarantine^16^, even though different studies have suggested that a major obstacle to compliance with household quarantine is concern over loss of income resulting from prolonged absence from work^17^. Around the world during the coronavirus outbreak, governments implemented economic relief plans to help the people 18–21, but this was not so in most SSA countries except for South Africa^18^. In Israel, compensation increased the compliance rate with self-quarantine from 57% to 94% demonstrating that providing people with assurances about their livelihoods during self-quarantine is an important component of compliance with public health regulations^19^. Since the unemployed did not show any association with compliance to other measures, it could mean that their motivation for isolation and quarantine were ‘partial’. They probably were motivated by lack of jobs to take them out or the fact that there was no associated cost. With a current mobile phone penetration rate of 75% in SSA^22^, the use of novel mobile cash transfer options such as mobile money should be considered by SSA governments. This channel can reach the informal sector with cash sustenance packages during a lock down, so as to improve on qurantine and self isolation in future pandemics or epidemics.

In this study, univariate analysis revealed that SSAs who lived in Diaspora were more likely to wear face mask and self-isolate during the pandemic compared with those who lived in their respective countries of origin. These findings could be attributed to the fact that that the messages, enforcement and the levels of compliance to public health measures mandated by government differ between those living in diaspora and people who lived in SSA and these would significantly affect the responses and attitudes of the participants in this study. In addition, their knowledge and exposure to matters related to COVID-19 in particular and pandemics in general will be different between Africans in diaspora and those who lived locally. Despite these differences, Africans still share similar characteristics and this was demonstrated by the loss of significant difference between the groups after adjusting for potential confounding factors.Our study also found that practice of hand washing and avoiding large gathering were optimal while over two-third of the participants wear face mask and about on-third complied with quarantine. These findings are similar to the study published in the Centers for Disease Control and Prevention's Morbidity and Mortality Weekly Report presented data that showed that nearly two third of the people surveyed complied with the use of face masks, maintaining physical distancing (79.5%) and about 86% avoided gatherings of 10 or more^23^. However, more people reported practicing self-isolation in their study compared with our finding (77% versus 40%), which could be attributed to the relief measures/assistance from the US government which encouraged people to stay more at home compared to most African countries where little, or no help came from the governments.

Although compliance with measures showed no patterned association with the regions, Central African countries were more likely to observe the government imposed measures which could be explained by the fact that they had experienced repeated outbreaks of the deadly Ebola virus since the seventies right up to recent times^24^ and therefore this improved on their ability to take public health measures and messaging more seriously. Moreover, the Central African region was an early epicentre of the COVID-19 pandemic in Africa, with Cameroon for example confirming its first case as early as 5th of March 2020^25^, thereby creating huge awareness and fear and thus encouraging early compliance with lockdown measures. In this study, females were more likely to comply with self-quarantine and isolation compared with males, which is similar to a recent study where females were more likely to take protective measures during the pandemic^23^. This finding may be related to the employment of more SSA women than males in the formal sector^26^ and with the shut down of the formal sector during the lockdown, females likely stayed at home, thus reported self-isolation.

Similar to a previous studies^27^,^28^ age was associated with compliance with most of the public health measures examined, with older respondents more likely to wear face mask, practice handwashing but less likelyo attend crowded gatherings, practice quarantine and self-isolation. This indicates that age is an important determinant of compliance with public health measures to control COVID-19.

This study has some limitations. First, the survey was only administered online and therefore may not have captured the opinion of non-internet users in the rural areas where the reach of the internet remains low 22. This may also have excluded respondents from the older people in SSA countries who are less likely to use the internet compared to younger ones^29^. Also, the survey was available only in English such that it may have been impossible for some citizens of francophone countries in the SSA to participate. Hence, the results may not generalize to all Sub-Saharan African populations. It is also possible that respondents from some SSA countries like Tanzania may have been affected by the lockdown as the citizens were refrained from giving out information regarding the pandemic, hence the wide variation in the response rate per region. We could not determine the response rate of this study due to the method of dissemination of the survey link (online platforms). Another limitation was the lack of incentives and therefore no assistance from online companies for distribution of the survey may have affected the reach to respondents. The strengths of this study include that, it is the first sub-regional analysis of African respondents with respect to the current COVID-19 pandemic and offers a unique perspective on the SSA countries' compliance with public health measures to contain and prevent the spread of the infection and thus provides a valuable contribution for future interventions across the region. Despite the limited participation of Africans that lived in dispaora, to the best of our knowledge, this is the first study to examine the willingnss to comply with public health measures among Africans that lived locally and those in Diaspora during the COVID-19 pandemic.

## Conclusions

Sub-Saharan African respondents in this study were compliant with the public health measures put in place by the respective governments to control the spread of COVID-19, despite their inadequate knowledge of the disease. While individual/community level control measures are as important as government actions, the governments of SSA will need to consider relief packages for their citizens in times like this to help improve on compliance during outbreaks of this nature. Overall, this study calls on the the SSA countries to consider certain sociocultural and economic solutions to help improve preparedness and response to future outbreaks.

## Paper context

What is known: Public health compliance in relation to other pandemic including influenza have been investigated in non-Sub-Saharan African population.

What the paper adds to the topic: This study provides first evidence on key factors associated with willingness to comply with the public health measures during COVID-19 in Sub-Saharan Africa.

What the implications of the paper: Findings will enable public health researchers to target sub-populations not willing to comply to public health measures for other COVID-19 waves and future pandemics.

Data availability statement. The data that support the findings of this study are available on request from the corresponding author, ULO.
